# Three-dimensional speckle tracking echocardiography and cardiac magnetic resonance for left ventricular chamber quantification and identification of myocardial transmural scar

**DOI:** 10.1007/s12471-016-0876-9

**Published:** 2016-08-18

**Authors:** M. F. A. Aly, S. A. Kleijn, R. F. Menken-Negroiu, L. F. Robbers, A. M. Beek, O. Kamp

**Affiliations:** 1 Department of Cardiology, and Institute for Cardiovascular Research (ICaR-VU), VU University Medical Center, Amsterdam, The Netherlands; 2 Department of Cardiology, University Hospital, Beni-Suef, Egypt

**Keywords:** Three-dimensional echocardiography, Speckle tracking, Cardiac magnetic resonance, Ventricular function, Myocardial scar

## Abstract

**Background:**

We compared three-dimensional speckle tracking echocardiography (3DSTE) and its strain to cardiac magnetic resonance (CMR) with delayed contrast enhancement for left ventricular (LV) chamber quantification and transmurality of myocardial scar. Furthermore, we examined the ability of 3DSTE strain to differentiate between ischaemic and non-ischaemic LV dysfunction.

**Methods:**

In 80 consecutive patients with ischaemic and 40 patients with non-ischaemic LV dysfunction, the correlations between LV volumes and ejection fraction were measured using 3DSTE and CMR. Global and regional 3DSTE strains and total or percentage enhanced LV mass were evaluated.

**Results:**

LV end-diastolic and end-systolic volumes and ejection fraction correlated well between 3DSTE and CMR (r: 0.83, 0.88 and 0.89, respectively). However, 3DSTE significantly underestimated volumes. Correlation for LV mass was modest (r = 0.59). All 3DSTE regional strain values except for radial strain were lower in segments with versus segments without transmural enhancement. However, strain parameters could not identify the transmurality of scar. No significant difference between ischaemic and non-ischaemic LV dysfunction was observed in either global or regional 3DSTE strain except for twist, which was lower in the non-ischaemic group (4.9 ± 3.3 vs. 6.4 ± 3.2°, *p* = 0.03).

**Conclusion:**

3DSTE LV volumes are underestimated compared with CMR, while LV ejection fraction revealed excellent accuracy. Functional impairment by 3DSTE strain does not correlate well with scar localisation or extent by CMR. 3DSTE strain could not differentiate between ischaemic and non-ischaemic LV dysfunction. Future studies will need to clarify if 3DSTE strain and CMR delayed contrast enhancement can provide incremental value to the prediction of future cardiovascular events.

## Introduction

Left ventricular (LV) chamber quantification and delineation of myocardial scar are clinically important diagnostic, therapeutic and prognostic parameters [1–3]. To date, cardiac magnetic resonance (CMR) is the reference imaging for volume measurement and its delayed contrast enhancement (DCE) is the clinical method to identify myocardial fibrosis [1–3]. Moreover, CMR is able to suggest the aetiology of fibrosis according to its myocardial distribution [4]. Nevertheless, echocardiography remains the first-line imaging technique for LV assessment due to its ease of use and wide availability. Three-dimensional speckle tracking echocardiography (3DSTE) is a promising technique towards clinical implication by overcoming the drawbacks inherent to two-dimensional echocardiography (2DE) and allowing more robust LV quantification in a highly automated fast analysis [5–8]. A good correlation between 3DSTE and CMR for quantification of LV volumes and ejection fraction (EF) has been reported, although it is known that LV volumes are significantly underestimated by echocardiography compared with CMR [7, 8]. Recently, reference values for 3DSTE were provided [9, 10]. Currently, there are limited data on the ability of 3DSTE strain to identify myocardial scar. Therefore, the current study tested the ability of 3DSTE strain to quantify myocardial scar, to act as a simple guide for revascularisation strategies as compared with CMR DCE. In addition, we evaluated its accuracy for measuring LV volumes and EF in a large cohort of patients.

## Methods

### Study population

We prospectively enrolled 153 consecutive patients with LV systolic dysfunction (EF < 50 % by 2DE) referred for CMR DCE to assess and quantify myocardial scarring. All patients underwent 3DSTE on the same day as the CMR study. Thirty-three patients were excluded: 5 due to irregular heart rhythm, 23 due to poor image quality (defined as ≥3 non-visualised segments, blurred blood-tissue interface of the endocardial border or the presence of stitching artefacts precluding the analysis, 20 with 3DSTE and 3 with CMR) and 5 due to very low volume rate with 3DSTE (≤11 volume per second). Of the remaining 120 patients, 80 had ischaemic and 40 had non-ischaemic heart disease. Ischaemic patients were defined by having a coronary angiography showing significant stenosis (>50 % stenosis in ≥1 major coronary artery) or a history of angina, myocardial infarction or coronary revascularisation. Non-ischaemic heart disease was idiopathic LV dysfunction in 36 (90 %), myocarditis in 3 (8 %) and amyloidosis in 1 patient (2 %). All subjects gave informed consent and the local ethics committee approved the study.

### CMR imaging and analysis

CMR scans were performed on a 1.5T scanner (Sonata or Avanto, Siemens, Germany). ECG-gated cine images were acquired using a breath-hold segmented steady-state free precession sequence. Per patient, 8–10 short-axis views were obtained, starting at the mitral annulus, covering the entire left ventricle. Ten to 15 minutes after injecting 0.2 mmol/kg of a gadolinium-based contrast, enhanced images were acquired in the same orientation as the cine images.

All data were analysed on a separate workstation using dedicated software (MASS v.5.1 2010-EXP beta, Medis, the Netherlands). Endocardial and epicardial contours were manually traced, including the papillary muscles and trabeculations inside the LV cavity. Enhanced regions were then determined after thresholding signal intensity at five standard deviations above the mean signal intensity of remote normal myocardium [11, 12]. All areas of hyperenhancement were quantified by computer-assisted planimetry on each short-axis image. Total infarct size was calculated by summation of all slice volumes of hyperenhancement and the total extent of hyperenhancement was expressed as the percentage of total LV mass. Segments with ≥50 % extent of hyperenhancement were classified as transmurally infarcted, while segments with <50 % were classified as non-transmurally infarcted [1]. LV volumes and EF were computed by planimetry of all short-axis images.

### Echocardiographic imaging and analysis

**Fig. 1:**
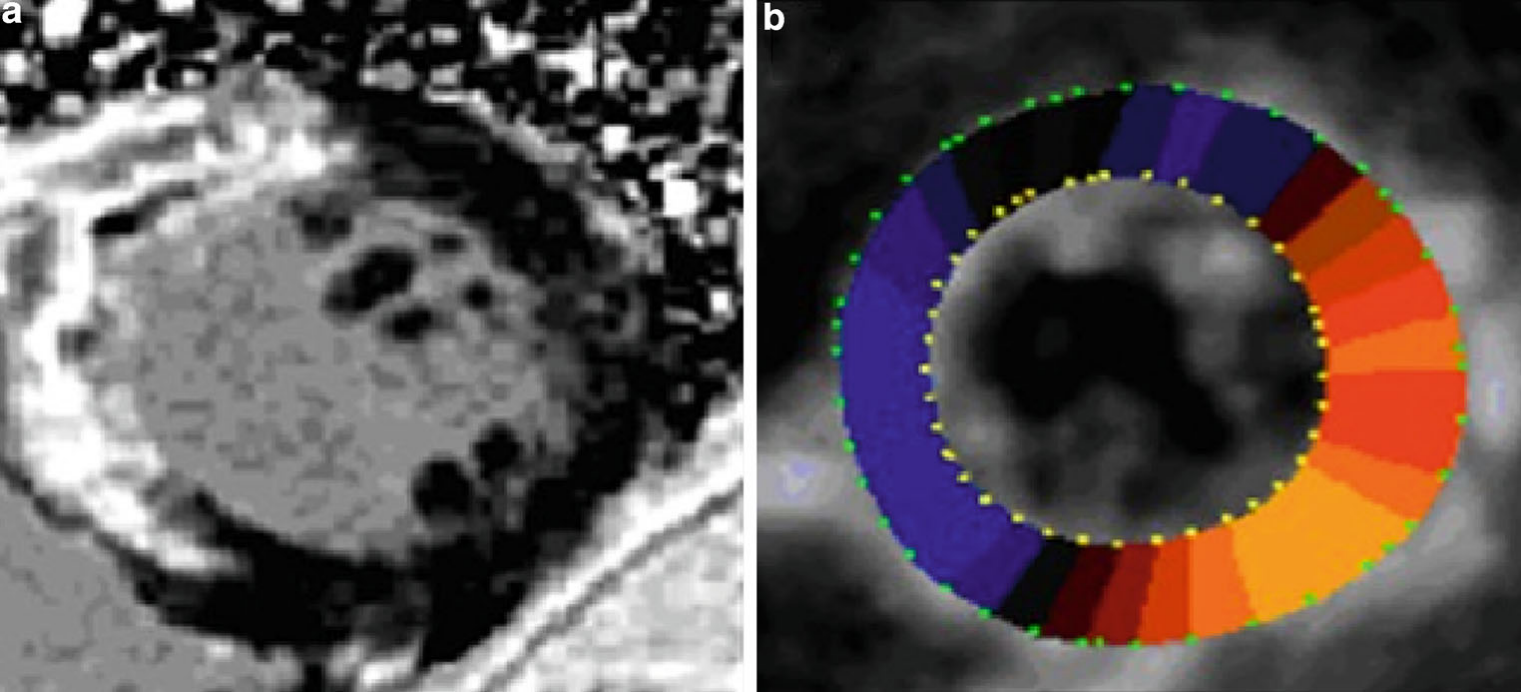
Cardiac magnetic resonance delayed contrast enhancement and three-dimensional speckle tracking echocardiography radial strain in a patient with transmural infarction. **a** Image of a patient with akinesia and transmural infarction of the septal and anterior walls (>50 % hyperenhancement) and (**b**) Colour-coded short-axis 3DSTE radial strain image at end-systole, radial strain is decreased as depicted by a blue colour overlay in a comparable region to the hyperenhancement in the CMR DCE, yet this area seems somewhat larger than the DCE one, involving the inferior wall as well. There is reddish colourisation in the other normal contracting segments with no hyperenhancement

3DSTE imaging was performed from an apical position using a commercial scanner (Artida 4D, Toshiba Medical Systems, Japan) with a fully sampled matrix array transducer (PST–25SX). Wide-angled acquisitions of 4 sub-volumes were acquired over 5 consecutive cardiac cycles during a single breath-hold, resulting in a mean temporal resolution of 23 ± 3 volume per second (range 17–26). Analysis of 3DSTE was done as previously described [6]. The 3DSTE strain data obtained included the three conventional strains (circumferential strain, longitudinal strain, and radial strain). In addition, two new strains, namely 3D strain and area strain, as well as twist were evaluated. 3D strain is a composite parameter of the three conventional strains. Area strain represents the endocardial area change at LV end-systole in relation to its original dimensions at end-diastole. Twist is defined as the maximal rotation seen in the most apical segment minus the maximal rotation seen at the most basal segment of the left ventricle. Fig. 1 depicts the difference between CMR DCE and 3DSTE radial strain in a patient with transmural infarction.

### Observer reliability

Observer reliability was assessed in 15 randomly selected patients for both 3DSTE and CMR DCE. Datasets were analysed for interobserver reliability by two experienced observers for each technique in a blinded fashion. Intraobserver measures were randomly performed on an average of 1 month apart.

### Statistical analysis

Continuous variables are presented as mean ± standard deviation, whereas categorical variables are presented as frequencies and percentages. Differences in continuous variables between the two groups were analysed using the Student’s *t*-test, while a Chi-square test or the Fisher’s exact test was used to analyse a difference in categorical variables as appropriate. Inter-technique comparisons between 3DSTE- and CMR-derived LV volumes, EF, and mass included linear regression and Bland-Altman analyses. The significance of differences between the two techniques was tested using paired *t*-tests. Reliability was assessed using the intraclass correlation coefficient (ICC). The clinical significance of ICC was interpreted as good if ICC ≥ 0.75. A *p* value < 0.05 was considered statistically significant. All data were analysed using SPSS version 20.0 (IBM SPSS Inc., Chicago, IL, USA).

## Results

### Baseline characteristics

The patient groups with ischaemic and non-ischaemic LV dysfunction were matched for age and body surface area. However, male gender, hypertension and diabetes mellitus were more frequent in ischaemic patients and the majority of positive DCE studies (89 %) were in the ischaemic patients (Table 1).

**Table 1:** Baseline and CMR characteristics of study population (*n* = 120)

Variable	Ischaemic patients *(n = 80)*	Non-ischaemic patients *(n = 40)*	*p* value
Age (years)	63 ± 12	59 ± 14	0.1
Men (*n*, %)	65, 79	27, 61	0.03
BSA (kg/m^2^)	1.9 ± 0.2	1.9 ± 0.2	0.6
Hypertension (*n*, %)	42, 53	18,45	<0.001
Diabetes mellitus (*n*, %)	20, 25	7, 18	<0.001
LVEDV (ml)^a^	202 ± 58	234 ± 73	0.009
LVESV (ml) ^a^	126 ± 58	154 ± 73	0.02
LVEF (%)^a^	40 ± 8	37 ± 9	0.2
LV mass (g) ^a^	110 ± 26	147 ± 50	<0.001
Positive DCE (*n*, %)^a^	71, 89	11, 28	<0.001
Enhanced LV mass (g)^a^	21 ± 15	6 ± 4	<0.001
Percentage enhanced LV mass (%)^a^	19 ± 11	4 ± 4	<0.001
Enhanced segments/patient (*n*)^a^	9.5	1.7	<0.001

^a^CMR data

Values are presented as mean ± SD or absolute number, percentage

*P* value < 0.05 is significant

*BSA* body surface area, *CMR* cardiac magnetic resonance, *DCE* delayed contrast enhancement, *LVEDV* left ventricular end-diastolic volume, *LVESV* left ventricular end-systolic volume, *LVEF* left ventricular ejection fraction,* LV* left ventricular

### Correlation between 3DSTE and CMR DCE for chamber quantification

**Fig. 2:**
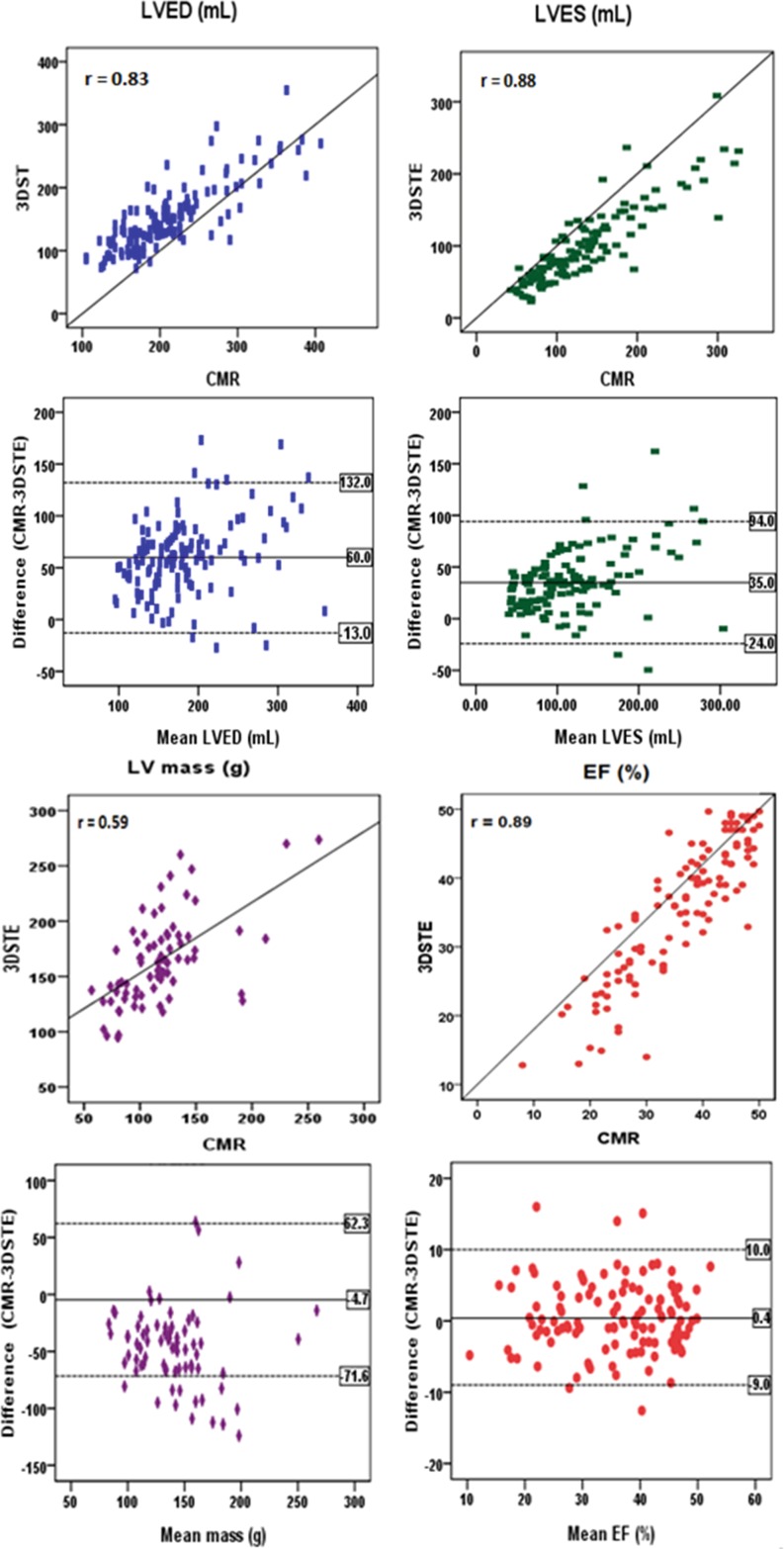
Comparison of left ventricular volumes, ejections fraction and mass by 3DSTE with CMR Results of linear regression (*top*) and Bland-Altman (*bottom*) analyses for each parameter. *r* Pearson’ correlation coefficient

For global chamber quantification in the whole study population, 3DSTE and CMR LVEF (39 ± 8 vs. 40 ± 7 %), LV end-diastolic (153 ± 54 vs. 213 ± 65 ml) and LV end-systolic volumes (98 ± 53 vs. 133 ± 64 ml) correlated well (r: 0.89, r: 0.83, and r: 0.88, respectively). Their correlation regarding LV mass (*n* = 82) (164 ± 39 vs. 117 ± 36 gm) was modest (r: 0.59). Despite a good correlation for LV volumes, 3DSTE significantly underestimated both volumes with relatively large biases (35–60 ml) and wide limits of agreement (30–67 ml), and significantly overestimated LV mass (bias is 47 g and wide limits of agreement: 38–54 g) and only LVEF has a small bias (0.4 %) and narrow limits of agreement (−0.5–1.3 %). Fig. 2 compares LV volumes, EF and mass by 3DSTE with CMR. For global 3DSTE strain, there were good correlations with LVEF by CMR, with the best correlations found for area stains and circumferential strain (r = −0.82 and −0.78, respectively) (Table 2).

**Table 2:** Correlation between global LV 3DSTE strain parameters and CMR in patients with positive delayed contrast enhancement (*n* = 82)

	CMR					
3DSTE	Enhanced LV mass (gm)		Enhanced LV mass (%)		LVEF (%)	
	*r*	*p* value	*r*	*p* value	*r*	*p* value
*All patients (n = 82)*						
LVEF (%)	−0.32	0.002	−0.29	0.009	0.88	<0.001
CS (%)	0.28	0.008	0.22	0.044	−0.78	<0.001
LS (%)	0.20	0.06	−0.21	0.06	−0.71	<0.001
RS (%)	−0.07	0.5	−0.01	0.92	0.51	<0.001
3DS (%)	−0.11	0.3	−0.01	0.92	0.51	<0.001
AS (%)	0.33	0.002	−0.29	0.009	−0.82	<0.001
Twist (°)	0.26	0.02	0.25	0.03	−0.25	0.3
*Ischaemic patients (n = 71)*						
LVEF (%)	−0.46	<0.001	−0.39	0.001	0.88	<0.001
CS (%)	0.38	0.001	0.32	0.006	−0.76	<0.001
LS (%)	0.27	0.02	0.29	0.02	−0.50	<0.001
RS (%)	−011	0.4	−0.08	0.5	0.44	<0.001
3DS (%)	−010	0.4	−0.29	0.02	−0.43	<0.001
AS (%)	0.44	<0.001	0.39	0.001	−0.83	<0.001
Twist (°)	0.24	0.06	0.28	0.03	−0.22	0.08
*Non-ischaemic patients (n = 11)*						
LVEF (%)	−0.28	0.4	0.11	0.7	0.86	<0.001
CS (%)	0.27	0.3	−0.01	0.97	−0.62	0.04
LS (%)	0.17	0.6	−0.16	0.6	−0.54	0.09
RS (%)	−0.04	0.9	0.29	0.4	0.69	0.02
3DS (%)	0.03	0.9	0.40	0.3	0.71	0.01
AS (%)	0.28	0.4	−0.05	0.7	−0.67	0.03
Twist (°)	−0.02	0.97	−0.05	0.9	−0.17	0.7

Data are expressed in Pearson’s correlation coefficient* (r)*

*P value* < 0.05 is significant

*LV* left ventricular, *3DSTE* three-dimensional speckle tracking echocardiography, *CMR* cardiac magnetic resonance, *LVEF* left ventricular ejection fraction,* CS* circumferential strain, *LS* longitudinal strain, *RS* radial strain, *3DS* three-dimensional strain, *AS* area strain

### Correlation between 3DSTE and CMR DCE for identification of myocardial scar

Global 3DSTE strains in ischaemic and non-ischaemic patients with positive DCE (*n* = 82) correlated poorly with either the total or the percentage enhanced LV mass. These correlations improved non-significantly when applied in the ischaemic group only and became worse in the non-ischaemic group (Table 2). After exclusion of 231 non-assessable segments by 3DSTE, which were primarily located in the anterior wall and apex (88 % of all uninterpretable segments), the remaining segments (*n* = 1689) were classified according to the result of CMR DCE as follows: group A with 0 % hyperenhancement (*n* = 864), group B with non-transmural hyperenhancement (1–50 %, *n* = 668), and group C with transmural hyperenhancement (51–100 %, *n* = 157). The majority of enhanced segments were in the ischaemic group (*n* = 757, 92 %). On this regional level, the correlations between 3D strains and the percentage of myocardial scar were poor as well, and this was true for both the segments with (r = 0.09 to 0.24) or without (r = 0.01 to 0.08) transmural hyperenhancement. By dividing the segments into three groups (basal, middle and apical) the correlations between different types of strain in each group and the percentage of myocardial scar were poor with better correlations for apical strains (radial strain: r = −0.14, *p* = 0.03; circumferential strain: r = 0.25, *p* < 0.001; longitudinal strain: r = 0.16, *p =* 0.01; 3D stain: r = −0.16, *p =* 0.01; area strain: r = 0.31, *p* < 0.001).

### Assessment of myocardial scar

**Fig. 3:**
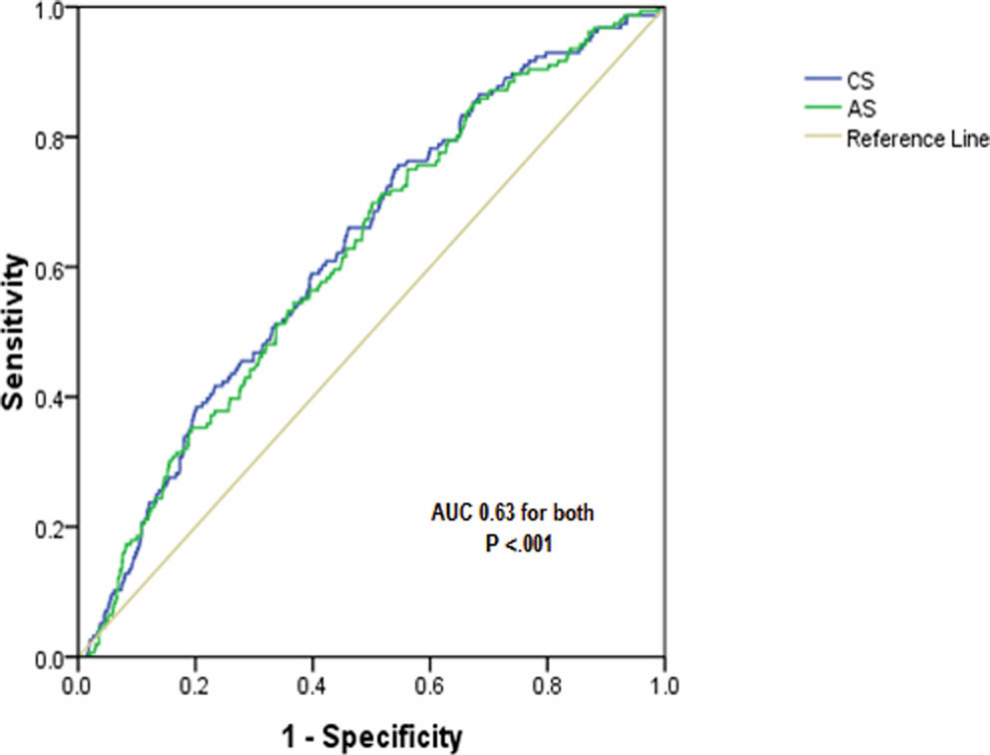
ROC curve analysis of segmental circumferential strain (CS) and area strain (AS) to differential between transmural and non-transmural segmental hyperenhancement

In segments showing hyperenhancement in ischaemic and non-ischaemic patients, 3DSTE could define lower values for circumferential strain and area strain in segments with non-transmural hyperenhancement compared with non-enhanced segments (*p* = 0.02 for both). In addition, it defined lower values for all strain components except radial strain in segments with versus those without transmural hyperenhancement (Table 3). Through receiver operating characteristic (ROC) curve analysis, the yielded area under the curve (AUC) for all strain parameters was low for differentiation of transmural from non-transmural enhanced segments (the best was 0.62 for each of circumferential strain and area strain as shown in Fig. 3 using ROC curve analysis of segmental circumferential strain and area strain to differentiate between transmural and non-transmural segmental hyperenhancement) as well as for differentiating non-transmural from non-enhanced segments (the best was 0.54 for circumferential strain and 0.53 for area strain). Similarly, on the level of basal and middle segments, strain values were similar in segments with and those without transmural scar; however, they were significantly lower in segments with transmural scar on the apical level (radial strain: 14.7 ± 9 vs. 18.6 ± 13, *p* = 0.004; circumferential strain: −14.6 ± 8 vs. −18.6 ± 10, *p* = 0.001; longitudinal strain: −8 ± 5 vs. −9.6 ± 7, *p* = 0.02; 3D strain: 15.4 ± 9 vs. 20 ± 14, *p* = 0.001; area strain: −18.7 ± 9 vs. −25.8 ± 12, *p* < 0.001, respectively). However, the yielded AUC for all strain parameters was low for differentiation of transmural from non-transmural enhanced segments, with circumferential stain and area strain performing best (r = 0.67 for both).

**Table 3:** Comparison between segmental 3DSTE strain values according to the extent of hyperenhancement (*n* = 82)

Variable	Non-enhanced (0 %) (A) *(n = 864)*	Non-transmural enhanced (<50 %) (B) *(n = 668)*	Transmural enhanced ≥50 % (C) *(n = 157)*	*p* value A vs. B	*p* value B vs. C
CS (%)	−20 ± 9	−18 ± 9	−15 ± 8	0.02	*<0.001*
LS (%)	−11 ± 7	−11 ± 7	−9 ± 6	0.07	*0.007*
RS (%)	24 ± 16	23 ± 17	20 ± 16	0.8	0.06
3DS (%)	26 ± 18	25 ± 17	22 ± 16	0.8	*0.03*
AS (%)	−28 ± 12	−26 ± 11	−22 ± 10	0.02	*<0.001*

*P* value < 0.05 is significant

*CS* circumferential strain, *LS* longitudinal strain, *RS* radial strain, *3DS* three-dimensional strain, *A* area strain

### Comparison between ischaemic and non-ischaemic patients using 3DSTE

There was no significant difference between the two groups regarding image quality, volume rate or heart rate, and no significant differences were found regarding LV volumes, EF and all types of global 3D strains. Only twist was significantly lower in the non-ischaemic compared with the ischaemic group; however, the AUC was low (0.55) to differentiate between the two groups (Table 4).

**Table 4:** 3DSTE global parameters in patients with ischaemic and non-ischaemic LV dysfunction (*n* = 120)

Variable	Ischaemic patients *(n = 80)*	Non-ischaemic patients *(n = 40)*	*p* value
3DSTE image quality (%)	^a^(63)/^b^(37)	^a^(60)/^b^(40)	0.8
Heart rate (bpm)	68 ± 11	70 ± 12	0.6
3DSTE volume rate (vps)	20 ± 3	21 ± 3	0.3
LVEDV (ml)	149 ± 53	160 ± 55	0.3
LVESV (ml)	95 ± 52	104 ± 57	0.4
LVEF (%)	39 ± 10	38 ± 11	0.7
LV mass (*n* = 82) (g)	152 ± 35	154 ± 35	0.8
CS (%)	−18 ± 7	−17 ± 9	0.8
LS (%)	−10 ± 4	−10 ± 4	0.9
RS (%)	21 ± 10	19 ± 10	0.2
3DS (%)	23 ± 10	19 ± 11	0.09
AS (%)	−26 ± 9	−25 ± 11	0.9
Twist (°)	6.4 ± 3.2	4.9 ± 3.3	0.03

^a^good image quality

^b^moderate image quality

*P value* < 0.05 is significant

*3DSTE* three-dimensional speckle tracking echocardiography, *LV* left ventricular, *LVEDV* left ventricular end-diastolic volume, *LVESV* left ventricular end-systolic volume, *LVEF* left ventricular ejection fraction, *CS* circumferential strain, *LS* longitudinal strain, *RS* radial strain, *3DS* three-dimensional strain, *AS* area strain

### Observer reliability

The intra- and inter-observer reliability for assessment of LV volumes, mass and EF using 3DSTE were excellent. However, intra-observer reliability was better than inter-observer reliability for assessment of different strain components. For the assessment of LV volumes, EF, mass, enhanced and percentage enhanced mass using CMR, both the intra- and inter-observer reliability were excellent (Table 5).

**Table 5:** Reliability of LV volumes and functions using 3DSTE and CMR (*n* = 15)

	Intra-observer			Inter-observer		
	Values 1	Values 2	*ICC	Values 1	Values 3	^*^ICC
3DSTE						
LVEDV (ml)	153 ± 50	150 ± 51	0.95	153 ± 50	147 ± 49	0.91
LVESV (ml)	97.5 ± 50	95 ± 50	0.95	97.5 ± 50	93 ± 51	0.90
LVEF (%)	39.4 ± 12	39.2 ± 12	0.93	39.4 ± 12	38.6 ± 12	0.90
LV mass (g)	140 ± 37	133 ± 33	0.83	140 ± 37	150 ± 38	0.77
Strain (%)	−7 ± 20.7	−8 ± 21.8	0.86	−7 ± 20.7	−8 ± 20.2	0.82
LS (%)	−10.1 ± 3.4	−9.8 ± 2.4	0.92	−10.1 ± 3.4	−11.1 ± 2.4	0.82
RS (%)	19.6 ± 9	18 ± 9.4	0.80	19.6 ± 9	17.1 ± 8.6	0.62
3D strain (%)	17.5 ± 6.5	18 ± 6.5	0.81	17.5 ± 6.5	20 ± 7.0	0.66
AS (%)	−30.5 ± 8	−29.5 ± 8	0.90	−30.5 ± 8	−31.9 ± 8	0.83
Twist (°)	5.3 ± 3.0	6.0 ± 2.8	0.90	5.3 ± 3.0	4.7 ± 2.6	0.70
CMR						
LVED (ml)	204 ± 60	200 ± 58	0.96	204 ± 60	199 ± 59	0.94
LVES (ml)	123 ± 58	120 ± 58	0.96	123 ± 58	119 ± 59	0.93
LVEF (%)	40 ± 12	39.8 ± 11	0.95	40 ± 12	39.5 ± 11	0.92
LV mass (g)	130 ± 42	125 ± 39	0.90	130 ± 42	122 ± 30	0.80
Enhanced LV mass (g)	9 ± 16.0	16.2 ± 8	0.92	9 ±16.0	15.7 ± 8	0.83
Percentage enhanced LV mass (%)	10 ± 13.5	9 ±13.8	0.95	10 ± 13.5	14.6 ± 11	0.83

*All *P* values <0.001. *P* value <0.05 is significant

*ICC* intra-class correlation coefficient, *3DSTE* three-dimensional speckle tracking echocardiography, *CMR* cardiac magnetic resonance, *LV* left ventricular, *LVEDV* left ventricular end-diastolic volume, *LVESV* left ventricular end-systolic volume, *LVEF* left ventricular ejection fraction, *CS* circumferential strain, *LS* longitudinal strain, *RS* radial strain, *3DS* three-dimensional strain, *AS* area strain

## Discussion

3DSTE represents a major innovation towards more comprehensive LV quantification, including principal indices as LV volumes and EF, but also parameters directly assessing global and regional myocardial function using strain. The aim of this study was to test the ability of 3DSTE to provide accurate and rapid chamber quantification and viability assessment as compared with CMR DCE.

### Correlation between 3DSTE and CMR for LV chamber quantification

Despite their good correlation, 3DSTE significantly underestimated both LV volumes and overestimated myocardial mass compared with CMR. The potential reasons for these known discrepancies were thoroughly discussed in our previous work [7]. Therefore, both 3DSTE and CMR are clinically applicable for measuring LV volumes but cannot be used interchangeably as is the case for most cardiac imaging modalities. The obtained good correlation and accuracy for LVEF between 3DSTE and CMR has been previously reported [7] and its accuracy and reproducibility was not affected by observer experience compared with the volumetric method [13]. This finding has important clinical consequences as LVEF is the most frequently performed echocardiographic assessment and many clinical decisions depend on its accurate measurement such as when to initiate cardiac medications, guide device therapy or perform cardiac surgery. These findings are in agreement with previous studies comparing both techniques in healthy subjects and cardiac patients [7, 8] and provide incremental value to currently used 2DE EF, which gave significantly higher values when compared with CMR [14]. Currently, 3DE assessment of LV volumes and EF is recommended over the use of 2DE, as it has been clearly demonstrated to provide more accurate and reproducible measurements [15].

### Correlation between 3DSTE strain and CMR DCE for identification of myocardial scar

3DSTE strain evaluates the functional consequences of myocardial fibrosis and CMR DCE evaluates its mere anatomical extent. For global 3DSTE strain, the agreement between global 3DSTE strains and CMR DCE was poor. These findings are in line with previous studies reporting that 2DSTE global longitudinal strain is markedly attenuated regardless of the extent of myocardial fibrosis as delineated by CMR DCE [16]. In addition, our results are similar to the results by Hayat et al. [17] who used the same 3DSTE machine and software and correlated 3DSTE strain with myocardial infarct size assessed by CMR in a much smaller number of ischaemic patients (*n* = 25). Although they stated that both are well correlated, their reported Pearson’s correlation coefficients were all <0.5, which should be interpreted as moderate to poor correlation from both a statistical and a clinical point of view. Furthermore, our findings are supported by a recent similar study in 58 ischaemic patients using 3DSTE equipment by a different vendor, which reported moderate to poor correlations for both global and regional 3DSTE strains [18].

For regional 3DSTE strain, previous studies using Doppler strain and 2DSTE in this regard have shown mixed results [19–21]. In the current study, the agreement between regional 3DSTE strains and CMR DCE was poor, for instance; strain values were not always close to zero in segments with complete transmural hyperenhancement and were low in non-enhanced segments. This controversy may be due to tethering from adjacent segments and the presence of other pathologies beyond fibrosis contributing to decreased strain, such as LV hypertrophy or hibernation [22]. Adequate alignment of segments between different imaging modalities as well as measurement artefacts and other technical issues such as differences in temporal and spatial resolution and algorithms of quantification may be additional factors. Similarly, CMR DCE is still prone to artefacts which can be falsely interpreted as hyperenhancement, although we used the five standard deviations (5SD) quantitative method which should largely exclude such artefacts. Despite these inaccuracies, some 3DSTE strains, namely circumferential strain and area strain, could differentiate between non-infarcted segments and segments with non-transmural infarction, but also between segments with non-transmural and transmural infarction, which is clinically more important for revascularisation decision. Despite these observations, all parameters had insufficient sensitivity and specificity for a meaningful clinical use.

### 3DSTE strain and detection of myocardial pathology

It is well established that the longitudinal myocardial mechanics are mainly governed by the subendocardial longitudinally arranged fibres and the circumferential mechanics are mainly dependent on the midmyocardial circumferentially arranged fibres [23]. Likewise, in myocardial infarction, DCE consistently involves the subendocardial layer with variable transmural extension, whereas it characteristically appears as intramyocardial midwall hyperenhancement in about one third of non-ischaemic cardiomyopathy [24]. Accordingly, it would be expected to find a difference in different types of 3D strains, particularly in longitudinal strain, in patients with versus patients without ischaemic LV dysfunction. However, in the current study, this was not established. This can be explained by the finding that a non-specific early decrease in LV longitudinal function through impairment of the subendocardial layer may occur in non-ischaemic cardiomyopathy as well [16, 25, 26]. Of note, since myocardial layers are variably affected by different disease processes, multi-layered assessment may be more useful. Previous studies using 2DSTE demonstrated better accuracy for the multilayer assessment compared with the assessment of the full wall thickness [19]. To date, only full thickness wall assessment is available with 3DSTE.

### Limitations

Current limitations of 3DSTE are the relatively low spatial and temporal resolution and unreliable acquisition in the presence of ab irregular heart rhythm, which lead to limited feasibility. In addition, the current lack of standardisation among different ultrasound machines and software packages provided by different vendors may preclude generalisation of our results [27]. However, two commonly used 3DSTE vendors had the same negative results [17, 18]. Although 3DSTE strain has been validated against CMR tagging [12], there is no true non-invasive reference technique to validate regional ventricular function, so its accuracy cannot currently be adequately established. A study to analyse the prognostic impact of both modalities, and to investigate the incremental value of 3D strain and percentage enhanced LV mass, compared with LVEF, for predicting future major cardiovascular events particularly in patients with ischaemic heart disease is therefore strongly recommended [28].

## Conclusion

3DSTE-derived LV volumes are underestimated compared with CMR, while measurement of LVEF revealed excellent accuracy. Functional impairment by 3DSTE strain does not correlate well with scar localisation or extent by CMR. In addition, 3DSTE strain could not differentiate between ischaemic and non-ischaemic LV dysfunction. Future studies will need to clarify whether 3DSTE strain and CMR DCE can provide incremental value to the prediction of future cardiovascular events.
